# The Use of Toothpastes Containing Different Formulations of Fluoride and Bioglass on Bleached Enamel

**DOI:** 10.3390/ma16041368

**Published:** 2023-02-06

**Authors:** Zeynep Ergucu, Inci Yoruk, Ayşegül Erdoğan, Hayal Boyacıoğlu, Robert Hill, Aylin Baysan

**Affiliations:** 1 Department of Restorative Dentistry, Faculty of Dentistry, Ege University, 35040 Izmir, Türkiye; 2 Ege University Application and Research Center for Testing and Analysis (EGE MATAL), Ege University, 35040 Izmir, Türkiye; 3 Department of Statistics, Faculty of Science, Ege University, 35040 Izmir, Türkiye; 4 Centre for Oral Bioengineering, Barts and the London School of Medicine and Dentistry, Queen Mary University, London E1 2AD, UK

**Keywords:** bioactive glass, bleaching, enamel, remineralisation, mineral content

## Abstract

Objectives: To investigate the application of toothpaste either containing calcium sodium phospho-silicate bioglass (NovaMin) or calcium fluorosilicate bioglass (BioMinF) on the surface mineral composition and morphology of enamel after bleaching procedure. **Methods:** Thirty extracted noncarious human teeth were allocated into five groups (n = 6). **Group 1:** Bleaching using 40% hydrogen peroxide (HP) and fluoridated toothpaste containing bioactive glass (1450 ppm fluoride). **Group 2:** Bleaching using 40%HP and toothpaste containing calcium fluorosilicate bioglass (540 ppm fluoride). **Group 3:** Bleaching using 40%HP and fluoridated toothpaste (1450 ppm fluoride). **Group 4:** Bleaching alone using 40%HP. **Group 5:** Negative control with distilled water alone. The surface morphology was evaluated using Scanning Electron Microscope (SEM) and Scanning Probe Microscope (SPM). The concentration of elements as atomic percentages were determined by X-ray Photoelectron Spectroscopy (XPS) and Energy-Dispersive X-ray Spectroscopy (EDS). **Results:** This laboratory-based study reported that SPM and SEM detected minor changes on the surfaces of all toothpaste-treated enamel samples (Groups 1–3) after 45 days. Bioactive glass deposits were observed on enamel surfaces in Groups 1 and 2, whilst the bleaching-alone samples (Group 4) had rough enamel surfaces. XPS reported that toothpaste containing calcium fluorosilicate bioglass (Group 2) had a high atomic% of calcium and phosphate, whilst silicon values were high in the toothpaste containing bioactive glass and 1450 ppm fluoride (Group 1) after bleaching procedure when compared to other groups (*p* < 0.05). In addition, EDS detected the highest %F in Groups 1, 2 and 5. **Conclusions:** Within the limitations of this laboratory-based study, there was no significant decrease in the Ca%, P% values and surface properties of enamel after the bleaching procedure following the use of different formulations of toothpastes for a period of 45 days. However, the Ca% and P% values were significantly high for the toothpaste containing calcium fluorosilicate bioglass (BioMinF) on the bleached enamel. **Clinical relevance:** The bleaching process can provide optimum aesthetic outcomes, but the effect of peroxides on hard tissues is still in question. Toothpastes containing different formulations of fluoride and bioactive glass might have the potential to prevent mineral loss on bleached enamel. However, further laboratory-based studies and controlled double-blind randomised clinical trials are required to interpret the effects of toothpastes with different fluoride and bioactive glass formulations on enamel surfaces following bleaching procedures.

## 1. Introduction

Tooth bleaching is one of the common management strategies for tooth discolouration and reported to be safe and effective when/if carried out by dentists [1]. Hydrogen peroxide is the active agent in most whitening products. This agent acts as a strong oxidising agent, producing reactive oxygen molecules and hydrogen peroxide anions. Hydrogen peroxide diffuses into enamel and dentine due its small molecule, where it decomposes into different active oxygen species under specific temperature, pH and light conditions [1]. The free radicals created could oxidise the conjugated structure of the chromophores [1], and this oxidation would promote morphological changes on enamel surfaces [2]. Therefore, the bleaching process has the potential to provide satisfactory aesthetic outcomes. However, the effect of peroxides on hard tissues is still in question [3,4,5,6].

The potential adverse effects of bleaching treatments might be minimised and mineral gain could be promoted. In this respect, remineralisation of enamel occurs by the deposition of calcium and phosphate ions on enamel surfaces in the presence of saliva [7]. In addition, toothpastes containing fluoride facilitate calcium and phosphate uptake from saliva and promote the formation of apatite on enamel surfaces [8,9]. In this respect, the use of fluoridated toothpastes on bleached enamel in relation to the mineral content was previously evaluated [10,11,12,13], but the process remains unclear.

Recently, bioactive glasses have been incorporated in toothpastes, dental varnish, prophy paste, cements, glass ionomers, acrylic and composite resins [14,15,16,17,18,19]. Interestingly, the bioactivity of these glasses is dependent on the compositional ratio of certain glass-forming system contents. Hench first developed bioactive glass in 1969 and this author suggested that these glasses dissolve/degrade in physiological-like fluids, forming hydroxycarbonated apatite (HCA) [14]. Subsequently, the bioactive glass (NovaMin developed by NovaMin Technology Inc., Alachua, FL, USA) based on this original 45S5 Bioglass (US Biomaterials Corp., Jacksonville, FL, USA) composition has been incorporated into a toothpaste for the management of dentine hypersensitivity. The mode of action was the precipitation of HCA onto the tooth surface and subsequent occlusion of dentinal tubules of [15], since bioactive glasses can boost their bioactivity in acidic environments and result in the releasing of calcium and phosphate ions onto the dental hard tissues that could promote remineralisation. In this regard, it was reported the long-term durability of HCA was limited in comparison to the formation of fluorapatite (FAp), which is resistant to acid attack. Therefore, fluoride (CaF_2_ and SrF_2_) was added to glasses rather than substituting them with calcium oxide (CaO) or strontium oxide (SrO), since this process was reported to maintain glass solubility, bioactivity and network connectivity, which are the main contributors to fast and chemically stable fluoroapatite formation. Biomin F was then introduced, which is an example of fluorine glass [13,16].

Bioactive glasses have been reported to be effective in remineralising early enamel carious lesions in comparison to other topical agents such as casein phosphopeptide-amorphous calcium phosphate (CPP-ACP) [13,14,15,16,17,18,19]. Bakry et al. [18] demonstrated that the calcium sodium phosphosilicate (45S5 bioglass, NovaMin; of nominal weight composition: 45% SiO_2_, 24.5% Na_2_O, 24.5% CaO, 6% P_2_O_5_) bioglass application was able to remineralise artificially-induced erosive enamel lesions which were stored in artificial saliva (pH: 6.5) for a period of two weeks. The authors indicated that the bioglass application resulted in the silica network of the 45S5 bioglass and this was thought to react with hydroxyl ions which were released from aqueous storage media and formed silanol compounds. The silica-gel rich layer was preserved during the first 24 h after the application. This process then played an important role in the formation of the calcium-phosphate rich layer. It was reported that as the particle reactions continued and the deposition of calcium and phosphorus complexes continued, this layer crystallised into hydroxycarbonate apatite.

Subsequently, the use of toothpaste containing bioglass (Sensodyne Repair & Protect, Haleon, Warren, NJ, USA; 45S5, NovaMin: 5.0% *w*/*w* active ingredient) during at-home bleaching procedures using 20% carbamide peroxide (Opalescence PF™, Ultradent Products Inc., South Jordan, UT, USA) showed promising results in a recent clinical study. It was reported that hypersensitivity reduced without any deteriorating effect. However, the study population was 24 for each group and the time period was seven days only [19], which is relatively short to assess the management of hypersensitivity.

There is still limited evidence to assess and compare the efficacy of different bioactive glass and fluoride formulations on bleached enamel. The aims of this laboratory-based study were therefore to investigate and compare the use of toothpaste either containing calcium sodium phosphosilicate bioglass (NovaMin) and fluoride or calcium fluorosilicate bioglass (BioMinF) on surface mineral composition and morphology of enamel after the use of 40% hydrogen peroxide bleaching. The null hypothesis is that there is no effect on the use of toothpaste either containing calcium sodium phosphosilicate bioglass (NovaMin) or calcium fluorosilicate bioglass (BioMinF) on surface mineral composition and morphology of enamel after the bleaching treatment. In addition, there is no difference between these toothpastes on the surface mineral composition and morphology of enamel after the bleaching process.

## 2. Materials and Methods

### 2.1. Sample Preparation

The local ethical approval was obtained prior to this study (Ethical application no. 18-12.1T/29). A total of 30 non-carious maxillary premolar human teeth extracted for orthodontic purposes, were randomly chosen from participants aged between 18 and 25 years old according to the inclusion and exclusion criteria. These teeth were stored in a mixture of 0.1% thymol with distilled water until their use (within a week) and this solution also changed once a week. Subsequently, these teeth were randomly allocated into five groups (Table 1). Each tooth was cleaned and polished using a polishing brush (PB-370, TPC Advanced Technology Inc., Industry, CA, USA) and water. The coronal parts of these teeth were cut from the cemento-enamel junction using a diamond disc (SD5122HP, Edenta, Switzerland) with water cooling and then divided into two from the bucco-palatal surfaces by the same method. These samples were measured using a caliper to create tooth blocks with an average of 3 mm thickness. Following this, the coronal parts of dental pulps were removed. Each sample was then stored in 0.1% thymol with distilled water at +4 °C until the test procedure.

### 2.2. Bleaching Procedure

In this study, the bleaching agent containing 40% hydrogen peroxide was used according to the manufacturer’s recommendations (Opalescence Boost 40%, Ultradent Products Inc., USA). The product was brought to room temperature before mixing. Subsequently, the gel was placed on the labial surfaces of each sample with a thickness of 0.5–1.0 mm and applied for 20 min. The same procedure was repeated three times. Finally, the agent was washed away using copious distilled water. After this procedure, the samples were kept in distilled water until toothbrushing procedures.

### 2.3. Toothpaste Treatment

Study groups with compositions of each toothpaste are described in Table 1. Toothpaste slurries with distilled water were used in 2:1 ratio for samples in Groups 1–3, whilst in Groups 4 and 5, toothbrushing with distilled water alone was carried out. A toothbrush with straight handle and medium-hard bristles was used in all groups (Basic Medium, Banat, Turkiye). Toothbrushing was carried out twice a day for a period of 45 days using a toothbrush simulating device (Toothbrush simulation ZM-3.4-SD Mechatronic, Izmir, Turkiye) designed to produce constant reciprocal movements (200 g force, at a frequency of 1.7 Hz for 10 s). The samples were left for two minutes prior to rinsing with deionised water [20]. These samples were stored at room temperature in distilled water for 24 hours until analyses were performed.

### 2.4. Scanning Probe Microscope (SPM)

After bleaching process and tooth brushing, each sample was first examined using the Scanning Probe Microscope (SPM, BRUKER Dimension Edge with ScanAsyst, Bruker, Heidelberg, Germany) to measure the surface topography and obtain the initial roughness values. SPM is a preferable technique for characterising the surface morphology of different materials at the nanoscale level. This method requires minimal specimen preparation/coating and is capable of evaluating the surface along the x, y and z axes. A very small probe (tip radius < 20 nm) is able to draw the profile of the surface morphology reproducing a quantitative x, y, z topographic map on the computer. The SPM image is a complete source of information about surface morphology [21].

The mid-third of the buccal surfaces for each sample was chosen in this study. Two-dimensional (2D) images were taken for each sample from the areas scanned at a width of 20 × 20 µm. Previously, the effects of bleaching on enamel microtopography were evaluated by scanning 15 × 15 µm areas [21]. However, in this study, large areas were chosen in order to get an adequate mapping of the morphological changes after treatment procedures. SPM software NanoScope Analysis (Bruker, San Jose, CA, USA) was used to obtain RMS (Rq- root mean squared), Ra (mean roughness) and Rmax (maximum roughness) values and SPM images.

Following this, atomic force microscopy (AFM), which is a type of SPM with a high-resolution three-dimensional (3D) imaging technique, was used for the characterisation of surface roughness [19,21,22,23]. In addition to the 2D images, all samples were also scanned using the AFM’s silicon tip (OTESPA-R3, Bruker, Santa Barbara, CA 93117, USA) with the tapping mode to provide 3D images from the same area for the optimal topographic visualisation. The tapping mode of the AFM has the advantage of avoiding any surface dragging effects of the tip on the surface [19].

### 2.5. X-ray Photoelectron Spectroscopy (XPS)

The X-ray Photoelectron Spectroscopy (XPS), which is a quantitative technique for chemical analysis, can measure elemental compositions of the chemical and electronic state of atoms on enamel surfaces. Following the scanning microscope examination, these samples were kept in a 37 °C vacuum oven for 24 h in order to carry out the XPS analyses. The polishing procedure was not required to avoid contamination or any misinterpretation. Eight different points from the mid-third of the buccal surfaces for each sample were selected and scanned. The presence of various elements [Calcium (Ca), Phosphorous (P), Nitrogen (N), Oxygen (O), Fluorine (F), C, Sodium (Na), Strontium (Sr), Tin (Sn), Silica (Si), Magnesium (Mg), Aluminum (Al), and Zinc (Zn)] were examined at these selected points. Analyses were then carried out using the Al Kα monochromatic X-ray source (1486.68 eV) and 300 µm X-ray spot size. Subsequently, data was recorded as the atomic percent (at%) values.

### 2.6. Scanning Electron Microscopy (SEM)

The samples were then coated with six nm-thick gold/palladium (Au/Pd) using the Leica EM ACE600 coating device (Leica Microscopes, Milton Keynes, UK). The standard drying process was not carried out since all samples were previously kept in a dessicator after the XPS analyses. After coating, samples were examined by Scanning Electron Microscopy (SEM, Thermo Scientific Apreo S LoVac SEM, Thermo Fisher Scientific, Waltham, MA, USA). The captured images were then evaluated and areas corresponding to the mid-third of the buccal surfaces for each sample were scanned. Images of the scanned areas at 1000×, 2500×, 5000× and 10,000× magnifications were recorded using the Everhart–Thornley detector (ETD).

### 2.7. Energy-Dispersive X-ray Spectrometry (EDS)

Scanning Electron Microscopy-Energy-Dispersive X-Ray Spectrometry (SEM-EDS) analysis was then performed to determine the mineral contents and examine the elemental compositions for each sample by connecting the EDS probe to a scanning electron microscope (Schottky Field Emission gun, Thermo Fisher Scientific, Waltham, MA, USA). The operating conditions were 20-KeV accelerating voltage, 10-nA beam current, and 30–45–s counting times with a 10 mm working distance. Under the given electron column conditions, 1–2 μm depth resolution was achieved with the EDS. The mid-part of the buccal surfaces with an area of 300 × 300 μm^2^ was scanned for each sample and the presence of Ca, P, N, O, F, C, Na, Sr, Sn, Si, Mg, Al, and Zn elements on the sample surfaces was examined. Mass percentages of the elements were then recorded.

### 2.8. Statistical Analyses

Descriptive statistics (arithmetic mean, maximum, minimum, standard deviation) were applied for the XPS and EDS data. Data presented normal distribution using the Shapiro-Wilk test. One-Way Analysis of Variance (ANOVA) was carried out to compare the groups, whilst Tamhane’s Post-Hoc Test was performed for the paired comparisons. SPSS 25.0 (IBM Corp., Armonk, NY, USA) package program was used for statistical analyses. The confidence interval was 95% (*p* < 0.05).

## 3. Results

This laboratory-based study reported that SPM and SEM detected minor changes on the surfaces of all toothpaste-treated samples after 45 days. Bioactive glass deposits were also observed in Groups 1 and 2 (SRP and BIO), whilst Group 4 (HP) had rough enamel surfaces. XPS reported that samples in Group 2 (BIO) had high atomic% of calcium and phosphate, whilst silicon values were high in Group 1 (SRP) when compared to other groups (*p* < 0.05). The highest %F was observed in Groups 1, 2 and 5 (SRP, BIO and UC).

### 3.1. SPM and SEM

**Figure 1:**
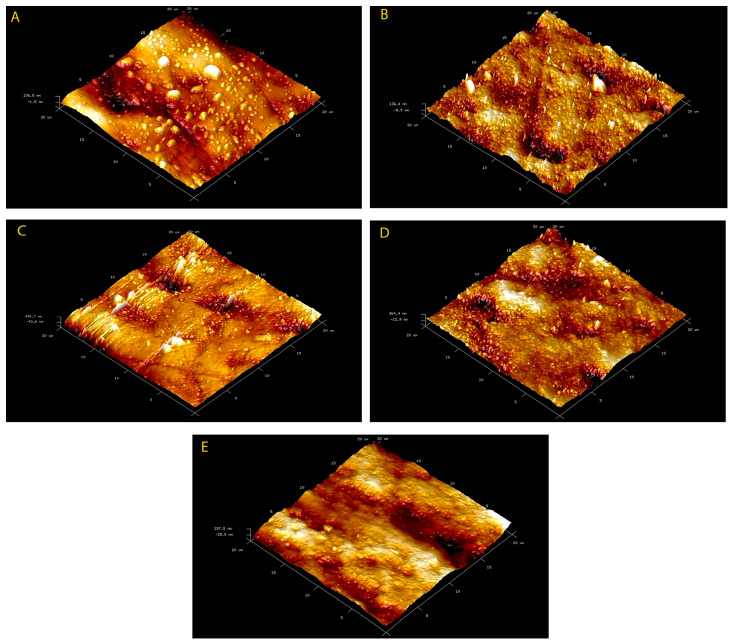
3D topographic SPM images after 45 days. (**A**) Group 1: Bleached enamel surfaces treated with standard fluoridated toothpaste with bioglass (NovaMin). (**B**) Group 2: Bleached enamel surfaces treated with toothpaste containing calcium fluorosilicate glass (BioMIN). (**C**) Group 3: Bleached enamel surface treated with toothpaste containing stardard fluoride alone. (**D**) Group 4: Bleached enamel surfaces with no toothpaste treatment. (**E**) Group 5: Enamel surface with no treatment.

**Figure 2:**
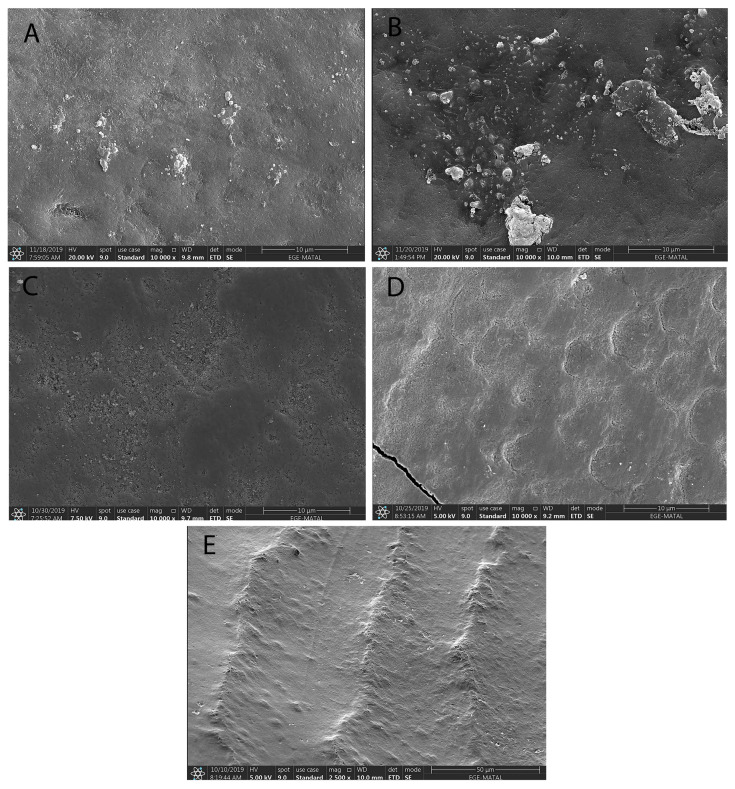
SEM images after 45 days. (**A**) Group 1: Bleached enamel surface treated with standard fluoridated toothpaste with bioglass (NovaMin). (**B**) Group 2: Bleached enamel surface treated with toothpaste containing calcium fluorosilicate glass. (**A**,**B**) precipitated layer of bioactive glass particles on the surfaces. (**C**) Group 3: Bleached enamel surface treated with standard fluoridated toothpaste. (**D**) Group 4: Bleached enamel surface with no toothpaste treatment showing some evidence of dissolved prism structures. (**E**) Group 5: Enamel surface with no treatment (×10,000 magnification).

Surface morphologies of all enamel samples were examined by the employment of SPM and SEM (Figure 1 and Figure 2). In the SPM and SEM analyses, minor changes such as increased porosity were detected in all toothpaste-treated groups (SRP, BIO, COL). Mineral deposits as well as bioactive glass deposits on the enamel surface were observed in Groups 1 and 2 (SRP and BIO). There were large bioactive glass particles (suggesting NovaMin) in Group 1 (SRP), whilst small particles were observed in Group 2 (BIO). Interestingly, smooth surfaces on the bleached samples were observed following the application of fluoridated toothpastes either with or without bioactive glass (Groups 1–3, SRP, BIO, COL). In this respect, the enamel samples in the bleaching-alone group (HP) had rough surfaces when compared to the other groups.

Roughness values were presented in Table 2. There were significant differences in the Rmax values for each group following a period of 45 days (*p* = 0.028). However, there were no statistically significant differences between the average roughness (Ra) and root mean square roughness (Rq) values (*p* > 0.05).

### 3.2. XPS and EDS

**Figure 3:**
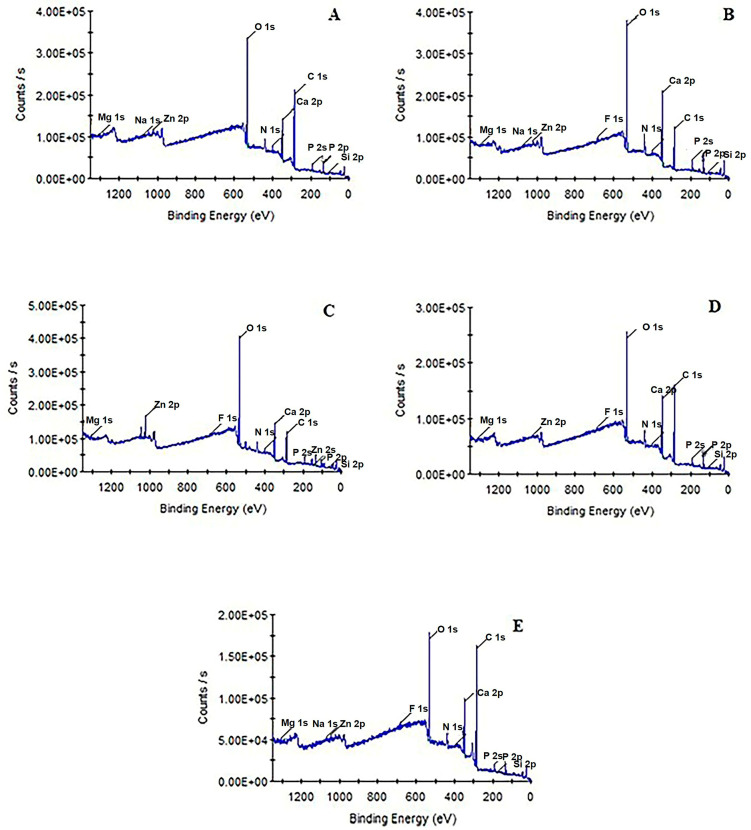
XPS survey spectra for enamel samples with different treatments except (**E**). (**A**) Group 1: Bleached enamel surface treated with fluoridated toothpaste containing bioglass (NovaMin). (**B**) Group 2: Bleached enamel surface treated with toothpaste with calcium fluorosilicate glass. (BioMIN) (**C**) Group 3: Bleached enamel surface treated with toothpaste containing standard fluoride. (**D**) Group 4: Bleached enamel surface with no toothpaste treatment. (**E**) Enamel surface with no treatment.

The presence of all elements in each group are depicted in the XPS survey spectra (Table 3). The analysed area using the XPS and EDS spectra for all samples are shown in Figure 3.

**Figure 4:**
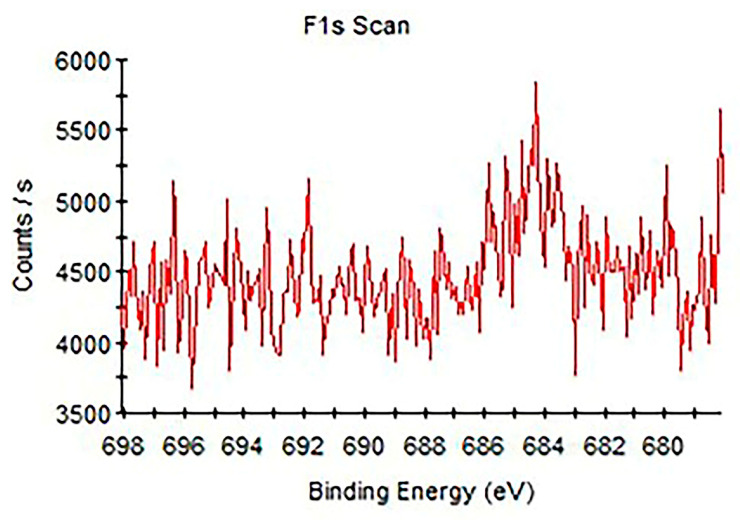
High resolution of XPS spectra of F 1 s for bleached enamel surface and subsequent fluoridated toothpaste containing bioglass (NovaMin) application for a period of 45 days (Group 1).

The results of XPS examination pointed to the development of fluorapatite. It should be noted that the existence of F 1 s signals at 684.0 eV indicated the existence of fluoride in Group 1 (SRP). Figure 4 shows the high-resolution XPS spectra of the F 1 s region. This spectrum displays a peak with binding energy of 684.1 eV which is similar to that reported for F in FAP (684.2 eV) in a previous study [24]. Samples in Group 2 (BIO) had high atomic% of phosphate and calcium. However, the silicon values were high in Group 2 (SRP) when compared to the other groups (*p* < 0.05). In addition, the amount of F% was indifferent between Groups 1–3 (SRP, BIO and COL). There was a lack of fluoride ions noted on the surface of samples in Group 5 (UC).

**Figure 5:**
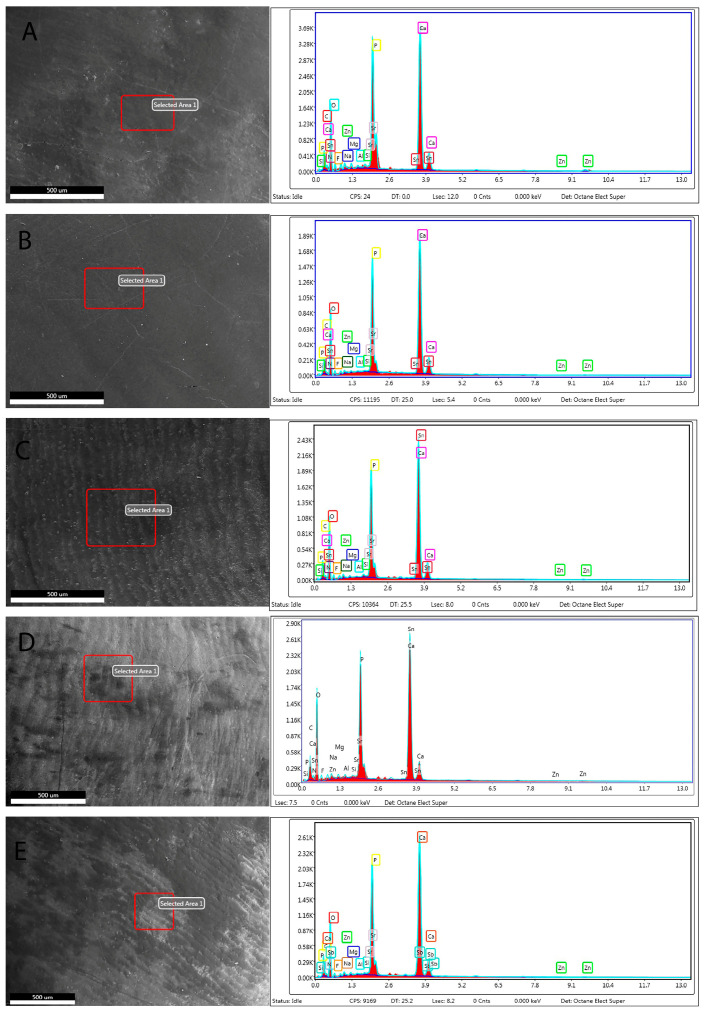
Representative SEM micrographs and EDS spectra of the analysed area for enamel surfaces except for (**E**). (**A**) Group 1: Bleached enamel surface treated with fluoridated toothpaste containing bioglass (NovaMin). (**B**) Group 2: Bleached enamel surface treated with toothpaste with calcium fluorosilicate glass (BioMIN). (**C**) Group 3: Bleached enamel surface treated with toothpaste containing standard fluoride. (**D**) Group 4: Bleached enamel surface with no toothpaste treatment. (**E**) Enamel surface with no treatment. In each measurement area (1 mm^2^), the intensity profile of the major elements (at.%) was analysed.

EDS is one of the most-used techniques for dental biomaterial chemical characterisation, since the probe depth is >1 μm [25]. There is no single technique that provides optimum answers to these analytical questions, and therefore different complementary techniques might be utilised to assess the efficacy of a dental material on enamel and dentine. EDS analyses were also performed to evaluate the chemical composition of all samples (Table 4). The advantage of the EDS analysis is to provide volumetric analysis (the depth is ~1 μm) compared to the XPS. Si% was also found to be the highest in Group 1 (SRP), whilst the highest Ca% was observed in Group 2 (BIO). In addition, the F% was mostly recorded in Groups 1 and 5 (SRP and UC) (Figure 5). Some outcomes differed from the XPS results, but this might be due to the fact that penetration depths in XPS analysis are ~ 10 nm when take-off angle did not change. Therefore, it should be noted that this technique provides superficial information.

## 4. Discussion

The effect of toothpastes containing different formulations of bioactive glass and fluoride on the surface changes of bleached enamel was evaluated in this laboratory-based study. There was no significant decrease in the Ca% and P% values and surface properties of bleached enamel following the use of different formulations of toothpastes for a period of 45 days. However, the Ca% and P% values were significantly high for the toothpaste containing calcium fluorosilicate bioglass (BioMinF).

Previous studies reported that the use of potassium nitrate and/or sodium fluoride reduced the intensity of tooth hypersensitivity after bleaching procedure [26,27,28]. Sodium fluoride might inhibit demineralisation by forming a calcium fluoride layer on enamel [29,30]. In this study, the increase in calcium loss would have led to high rates of the F% value in the bleaching-alone group; however, this does not mean that the samples in this group would contain more fluoride ions when compared to other groups. It could be speculated that this increase might be due to the fluoride content of the bleaching agent and/or the cumulative effect of fluoride ions within extracted teeth, since the fluoride ions in enamel could be detected at the surface.

In the XPS analysis, the amount of F% was indifferent in samples treated with different formulations of toothpastes following the bleaching process. Interestingly, the lack of fluoride ions was noted on the surfaces of enamel in the negative control group, i.e., samples received neither bleaching agent nor toothpastes. However, the EDS analysis reported the high fluoride levels in this group. This might be either due to the sensitivity variations of each technique, different measurement depths or the cumulative effect of fluoride ions within the extracted teeth. In addition, the XPS determines the elemental composition of the surface. Therefore, fluoride on the enamel surface might not have been detected using this technique. However, the EDS provides detailed information by detecting fluoride ions within the enamel surface (1–2 µm).

In both XPS and EDS analyses, the high percentage of Si was seen in the group using the standard fluoridated toothpaste containing bioglass (NovaMIN), and the differences between other groups were also significant. This bioactive glass contains silica-oxide (45 wt%, SiO_2_), and therefore the Si detected on the enamel surface could be from the ingredients of the toothpastes. This element was detected on the outer surface of the bleached enamel samples. While interpreting the differences encountered, it should be taken into consideration that these analyses were performed in randomly selected regions of the middle third of the sample. The depths of the analysed samples for chemical analysis vary according to the different techniques used in this study. Some techniques could provide information on the outer monolayer (<1 nm), while others would detect several nanometers depth. X-ray photoelectron spectroscopy (XPS) provides information from <1 nm by adjusting the take-off angle to several micrometers with etching of the surface. There are relatively few examples of surface analyses using XPS in dentistry [31,32,33]. The XPS has been used for the analysis of enamel by assessing the effects of various treatments such as argon laser irradiation on enamel [32] and effects of exposure to different chemical agents such as alkaline solutions with and without surfactants, as well as organic plaque and pellicle [34].

In real-life conditions, saliva would provide an effective environment for mineral gain after the bleaching procedure. However, the efficacy of saliva was not investigated in this study. Therefore, the effect of these toothpastes might not be clinically significant. Further laboratory-based studies and controlled double-blind randomised clinical trials are required to interpret the effects of bioactive glasses on enamel surfaces following the different bleaching procedures.

The bleaching procedure is still considered to be controversial. Although there were no surface changes in some studies, the non-uniform morphological changes on the enamel surface were observed after bleaching [34,35,36,37]. Pinto et al. [38] evaluated the effects of six different bleaching treatment (Whiteness Perfect—10% carbamide peroxide, Colgate Platinum—10% CP, Day White 2Z—7.5% hydrogen peroxide, Whiteness Super—37% carbamide peroxide, Opalescence Quick—35% carbamide peroxide and Whiteness HP—35% hydrogen peroxide) on enamel and these authors reported there was a decrease in the surface microhardness and increase in the surface roughness, especially following the use of 35% hydrogen peroxide. The SEM examinations of the bleached samples revealed the damaging effects such as interprismatic dissolution and erosion. In this current study, the SEM images also depicted dissolved prism structures in the bleached samples with no toothpaste treatment.

In addition, slight roughness and increased porosity were observed in all samples. In the SPM and SEM analysis, minor changes such as increased porosity were detected in the groups using the toothpastes with different formulations of bioglass and fluoride. Mineral deposits, including the bioactive glasses on the enamel surfaces, were also reported. This might be due to the interaction of Ca, P, Na and bioactive glass particles in the toothpastes. It should also be noted that there might be a possible ion reservoir for remineralisation in the HP-affected areas. Interestingly, large bioglass particles were also noticed in the standard fluoridated toothpaste with bioglass (NovaMIN) since the particle sizes for this glass are ~18 μm, compared to the BioMIN group, which had small bioglass particles (D50 of 6 μm). The SEM/EDS analysis also indicated a “repair effect” on the roughness of the demineralised enamel surface following the use of toothpaste containing calcium fluorosilicate glass (BioMIN), which was consistent with the previous studies [39,40,41]. On a separate note, Rotstein et al. [42] investigated the effects of six different bleaching agents on dental hard tissues by histochemical analysis and reported that hydrogen peroxide could affect the structure of dental hard tissues by changing the calcium concentrations in the tooth.

Fluoride applications are proven to manage dental caries by inhibiting enamel and dentine demineralisation, enhancing remineralisation by inhibiting bacterial enzymes [43]. An important factor is the formation of fluorapatite (FAp), which is acid resistant in comparison to the hydoxyapatite and carbonate-rich hydroxyapatite. In recent studies, bioactive glasses were found to be effective in enamel remineralisation [10]. In this respect, Vieira-Junior et al. [10] investigated the effects of different toothpastes on enamel surface prior to the bleaching procedure using 35% hydrogen peroxide, and these authors reported that toothpaste containing bioactive glass (NovaMin) was beneficial in minimising the negative bleaching effects related to mineral loss and changes in enamel topography. Subsequently, the use of different fluoridated toothpastes, either with bioglass or arginine carbonate, was investigated prior to bleaching with 35% hydrogen peroxide on the mineral content and surface morphology of enamel [44]. The EDS analysis showed that 35% hydrogen peroxide increased the loss of calcium and phosphate in enamel; however, the authors stated that fluoridated toothpastes with either bioactive glass or arginine carbonate were effective in protecting the bleached enamel against mineral loss.

In this study, bleaching procedure and subsequent toothpaste applications affected the enamel calcium and phosphate contents in accordance with the current literature. The results obtained by XPS and EDS analyses both confirmed that the mineral composition of the enamel surface was affected after bleaching with 40% hydrogen peroxide, i.e., Ca% and P% loss were detected. Although these results were not statistically significant, the findings from the previous studies in terms of mineral loss were consistent with this study. Gjorgievska et al. [12] previously reported that NovaMin particles increased Ca% and P% concentrations on the enamel surface and these authors also concluded that demineralised enamel surface could be remineralised following the use of toothpaste containing bioglass.

In addition, Bakry et al. [40] investigated the remineralisation of early artificial carious lesions and reported that toothpaste containing calcium fluorosilicate glass (BioMIN) was effective in remineralising the demineralised enamel lesions by allowing calcium and phosphate to penetrate into the porous sub-surface enamel lesion. However, a high concentration fluoride gel remineralised only the outer surface of the enamel. It should be noted that the toothpaste containing calcium fluorosilicate glass could play a role in the formation of fluorapatite since the concentration of fluoride within the glass is crucial to avoid the formation of fluorite. The adequate amount of fluorine inclusion into the bioactive glass would lead to the development of fluorapatite (FAP), which is chemically more stable than hydroxyapatite or carbonated hydroxyapatite. Due to the remineralisation effect, different bioactive glasses have extensively been investigated [12,13,15,18,19,20,39,40,41]. Bioactive glass, which is made up of amorphous sodium-calciumphosphosilicate, is a highly reactive material in an aqueous environment such as saliva in the oral cavity. Sodium ions from the bioactive glass particles readily react with hydrogen cations (in the form of H_3_O^+^) from saliva inducing the release of calcium and phosphate ions from the glass [39].

In this laboratory-based study, one of the limitations is the lack of representation of the oral cavity such as the absence of saliva, dental plaque, and salivary pellicle. It should be noted that in vitro models are unable to accurately represent the conditions that exist in the oral cavity. These models, on the other hand, could be used to assess new agents that are deemed to promote remineralisation prior to translational studies. Therefore, randomised controlled trials are required to assess the remineralisation effect of toothpastes containing different formulations of bioglass and fluoride on bleached enamel.

## 5. Conclusions

The tooth bleaching procedure can cause surface changes and mineral loss on enamel. The type of remineralisation agents in toothpastes plays a vital role in repairing the mineral content of enamel. The use of toothpastes containing different formulations of bioactive glass and fluoride resulted in mineral gain on the bleached enamel when compared to a standard toothpaste containing 1450 ppm fluoride alone. Interestingly, the toothpaste containing calcium fluorosilicate bioglass (BioMinF, 540 ppm fluoride) resulted in high elemental levels of calcium and phosphate on bleached enamel when compared to the toothpaste containing standard fluoride (1450 ppm) and bioactive glass (NovaMIN).

## Figures and Tables

**Table 1:** Study groups and compositions of each toothpaste.

Groups	Toothpaste	Treatment/Bleaching	Manufacturer	Composition
Group 1 SRP	Sensodyne Repair and Protect	Bleaching using 40% hydrogen peroxide (three times for 20 min each application)	Haleon Group of Companies, Weybridge, Surrey, UK	Glycerin, polyethylene glycols (PEG-8), Silica, Calcium Sodium Phosphosilicate (NovaMin), Sodium Lauryl Sulfate, Sodium monofluorophosphate, Aroma, Titanium Dioxide, Carbomer, Potassium, Acesulfame, Limonene, 1450 ppm Sodium Fluoride
Group 2 BIO	BioMinF	Bleaching using 40% hydrogen peroxide (three times for 20 min each application)	BioMin, London, UK	Glycerin, Silica, PEG 400, FluorocalciumPhosphoSilicate, Sodium Lauryl Sulphate, Titanium Dioxide, aroma, Carbomer, Potassium Acesulfame, 540 ppm fluoride
Group 3 COL	Colgate Total	Bleaching using 40% hydrogen peroxide (three times for 20 min each application)	Colgate Palmolive, Morristown, TN, USA	Water, Hydrated Silica, Glycerin, Sorbitol, PVM/MA Copolymer, Sodium Lauryl Sulphate, Flavour, Cellulose Gum, Carrageenan, Sodium Hydroxide, 1450 ppm Sodium Fluoride, Sodium Saccharin, Triclosan, CI 77891
Group 4 HP	Bleaching alone	Bleaching using 40% hydrogen peroxide (three times for 20 min each application)	Opalescence Boost, South Jordan, UT 84095, USA	
Group 5 UC	Distilled water alone	Unbleached control with no treatment	-	-

**Table 2:** Roughness values (Mean ± SD) for each group.

Roughness (µm)	Group 1 (SRP)	Group 2 (BIO)	Group 3 (COL)	Group 4 (HP)	Group 5 (UC)
Ra	0.06 ± 0.02	0.07 ± 0.03	0.07 ± 0.02	0.09 ± 0.03	0.05 ± 0.02
Rq	0.07 ± 0.03	0.08 ± 0.03	0.09 ± 0.03	0.12 ± 0.04	0.07 ± 0.03
Rmax	0.63 ± 0.30	0.85 ± 0.40	1.01 ± 0.26	1.2 ± 0.30 *	0.6 ± 0.23

* statistically significant (*p* < 0.5).

**Table 3:** Elemental composition (atomic %, Mean ± SD) of enamel samples using the XPS.

Elements	Group 1 (SRP)	Group 2 (BIO)	Group 3 (COL)	Group 4 (HP)	Group 5 (UC)
Si	5.42 (±3.16)	2.73 (±1.46)	2.07 (±0.93)	1.68 (±0.78)	1.28 (±0.46)
C	36.52 (±3.72)	36.17 (±8.83)	44.6 (±9.01)	56.46 (±12.06)	52.56 (±7.5)
N	2.02 (±0.98)	2.84 (±1.28)	1.45 (±0.54)	1.18 (±0.47)	3.48 (±0.88)
O	36.07 (±4.31)	34.54 (±5.54)	29.94 (±6.32)	25.21 (±6.71)	27.38 (±4.4)
F	0.83 (±0.3)	0.93 (±0.25)	0.84 (±0.18)	0.84 (±0.34)	˂0.05
Na	2.80 (±2.02)	1.20 (±1.35)	0.99 (±1.29)	1.87 (±1.68)	1.86 (±1.77)
Mg	0.55 (±0.2)	0.34 (±0.17)	0.38 (±0.17)	0.66 (±0.31)	0.41 (±0.16)
Al	˂0.05	˂0.05	˂0.05	˂0.05	˂0.05
Sr	1.26 (±0.42)	1.62 (±0.52)	1.40 (±0.45)	0.95 (±0.4)	0.98 (±0.32)
P	5.89 (±1.59)	7.20 (±1.95)	5.85 (±2.06)	4.41 (±1.63)	4.48 (±1.33)
Sn	˂0.05	0.37 (±0.77)	0.31 (±0.94)	0.06 (±0.4)	0.40 (±0.59)
Ca	8.20 (±2.28)	10.49 (±3.19)	9.66 (±2.97)	7.69 (±2.54)	7.33 (±1.80)
Zn	1.37 (±0.40)	0.43 (±0.21)	0.31 (±0.14)	0.41 (±0.26)	0.35 (±0.17)

**Table 4:** Elemental composition (atomic %, Mean ± SD) for each group after 45 days using EDS.

Elements	Group 1 (SRP)	Group 2 (BIO)	Group 3 (COL)	Group 4 (HP)	Group 5 (UC)
Si	0.20 (±0.03)	0.13 (±0.06)	0.19 (±0.10)	0.16 (±0.05)	0.09 (±0.03)
C	14.43 (±3.52)	12.88 (±3.81)	11.53 (±3.67)	19.6 (±3.76)	21.60 (±8.52)
N	7.15 (±0.73)	5.20 (±0.81)	5.52 (±1.21)	9.24 (±1.60)	6.23 (±2.08)
O	43.95 (±5.38)	46.45 (±7.89)	49.26 (±7.54)	48.52 (±5.51)	45.79 (±6.14)
F	3.65 (±0.32)	2.40 (±0.38)	2.34 (±0.35)	3.93 (±0.27)	2.11 (±0.39)
Na	0 (±0.002)	0 (±0.003)	0 (±0.003)	0	0
Mg	0.39 (±0.19)	0.22 (±0.17)	0.28 (±0.20)	0.50 (±0.11)	0.31 (±0.17)
Al	0.15 (±0.11)	0.11 (±0.08)	0.13 (±0.09)	0.20 (±0.06)	0.12 (±0.11)
Sr	0.03 (±0.01)	0.03 (±0.02)	0.03 (±0.02)	0.04 (±0.01)	0.05 (±0.05)
P	11.17 (±1.86)	11.02 (±2.16)	10.46 (±2.30)	6.87 (±1.51)	8.69 (±3.31)
Sn	0.03 (±0.02)	0.04 (±0.03)	0.05 (±0.02)	0.01 (±0.01)	0.05 (±0.08)
Ca	18.73 (±4.30)	21.45 (±6.83)	20.08 (±7.42)	10.88 (±3.17)	15.39 (±6.88)
Zn	0.08 (±0.06)	0.07 (±0.05)	0.14 (±0.31)	0.04 (±0.03)	0.03 (±0.03)

## Data Availability

The data that support the findings of this study are available from the corresponding author, Dr. A Baysan, upon request.
